# Do sexually transmitted infections exacerbate negative premenstrual symptoms? Insights from digital health

**DOI:** 10.1093/emph/eoy018

**Published:** 2018-07-03

**Authors:** Alexandra Alvergne, Marija Vlajic Wheeler, Vedrana Högqvist Tabor

**Affiliations:** 1 School of Anthropology and Museum Ethnography, University of Oxford, Oxford, UK; 2 Clue by BioWink GmbH, Adalbertstraße 7-8, Berlin, Germany; 3 2018 BOOST THYROID BY VLM HEALTH UG, Pufendorfstrasse 7, Berlin, Germany; 4 Stanford-SPARK, Berlin, Germany

**Keywords:** reproduction and hormones, evolutionary immunobiology

## Abstract

**Background and objectives:**

The underlying reasons why some women experience debilitating premenstrual symptoms and others do not are largely unknown. Here, we test the evolutionary ecological hypothesis that some negative premenstrual symptoms may be exacerbated by the presence of chronic sexually transmitted infections (STIs).

**Methodology:**

34 511 women were recruited through a digital period-tracker app. Participants were asked: (i) Have you ever been diagnosed with a STI? (ii) If yes, when was it, and were you given treatment? Those data were combined with longitudinal cycle data on menstrual bleeding patterns, the experience of pain and emotions and hormonal contraceptive use.

**Results:**

865 women had at least two complete menstrual cycle data and were eligible for analysis. Before diagnosis, the presence of an infection predicts a ca. 2-fold increase in the odds of reporting both headache, cramps and sadness during the late luteal phase and sensitive emotions during the wider luteal phase. After diagnosis, the odds of reporting negative symptoms pre-menstrually remain unchanged among STI negative individuals, but the odds of reporting sensitive emotions decrease among STI positive individuals receiving a treatment. No relationships between STIs, pain and emotions are observed among hormonal contraceptive users.

**Conclusions and implications:**

The results support the idea that a negative premenstrual experience might be aggravated by the presence of undiagnosed STIs, a leading cause of infertility worldwide. Caution is warranted in extrapolating the results as the data are self-reported, inflammatory levels are unknown and the tracker is biased towards recording negative premenstrual symptoms among Western individuals.

## BACKGROUND AND OBJECTIVES

Premenstrual syndrome (PMS), a chronic condition experienced by women before their menses and characterized by >200 negative physical and psychological symptoms (PMSx) including anxiety, depression, pelvic pain and headaches [1, 2], is common yet poorly understood [3]. In Western contexts [3] and in China [6], ca. 80% of fertile women experience one or several symptoms and for 3–8% of naturally cycling women, the experience of the pre-menstrual period is so debilitating that it is categorized as a premenstrual dysphoric disorder (PMDD) that requires medical attention [7, 8]. Therapies considered to be effective include anti-depressant medication [4], drospirenone-containing combined oral contraceptives [5] and cognitive behavioural therapy [3]. However, mechanisms for the onset of PMS and PMDD remain uncertain [5]. There are currently two inter-linked theories for explaining diversity in the experience of PMS: (i) an abnormal response to progesterone and (ii) lower levels of allopregnanolone, a neuroactive metabolite of progesterone with antidepressant action [3]. While biomedical research views premenstrual changes as the mark of a hormonal and psychological disease rather than a natural event, an evolutionary perspective points to the fundamental dialogue between the immune and neuroendocrine systems for understanding the health of living organisms [6].

Evolutionary approaches to the menstrual cycle conceptualize both menstruation and PMS as by-products of the evolution of the immune system in female bodies [4, 5] (see also [13, 14] for other evolutionary views). In menstruating species, growing evidence suggests that the uterus has evolved to be ‘choosy’, i.e. avoid maternal investment in compromised embryos [7–9]. This process, referred to as cyclic and spontaneous decidualization of the endometrium [10], takes place in the luteal phase and is underpinned by a bi-phasic immune response that optimizes the balance between embryo selectivity and embryo receptivity. In non-conceptive cycles, the ‘preparation’ of the endometrium is followed by menstruation, a massive inflammatory event necessary for the regeneration of the endometrium [10]. Thus, successful reproduction is critically dependent on the menstrual regulation of immunity, which is partly achieved through the actions of progesterone and oestrogen [11], two sex hormones produced by the ovaries (in non-conceptive cycles) and for which there are receptors on many immune cells [17]. As a result of hormonally induced shifts in immune function during the menstrual cycle, female health is expected to be cyclical [12] and premenstrual changes are expected to be a normal part of women’s experience. Although results are somewhat mixed, there is accumulating evidence that disease vulnerability, susceptibility and exacerbations do vary across the menstrual cycle [12].

Recent research has queried whether PMS can be understood as an ‘inflammatory disease’ [13, 14]. At first glance, such wording reinforces the negative cultural construction of premenstrual changes as pathological and fail to recognize that the premenstrual phase can be empowering [15]. However, PMS is also a lived experience and there is evidence that women do indeed report negative symptoms both in sociocultural contexts where premenstrual changes are seen as normal (e.g. in India and China where pain, fatigue and water retention are reported) and in Western contexts where they are seen as pathological (e.g. in the US or in Europe where negative mood symptoms are recorded) [16]. Thus, the underlying causes of PMS remain to be deciphered and the possibility that diversity in women’s experience of the premenstrual phase is, at the physiological level, underpinned by differences in levels of inflammation, is intriguing.

A study among 2 939 US women aged 42–52 years found that high-sensitivity C-reactive protein, a common marker of systemic inflammation, was associated with some PMSx (mood, cramps and weight gain) but not with others (headache and breast pain)[14]. Another study among 277 US women aged 18–30 suggested that pro-inflammatory factors, which result in sickness behaviour, depression [17] and impaired social interactions through immune-to-brain-signalling communication pathways [18], are elevated in women experiencing PMS [13]. However, the luteal-phase administration of progesterone aimed at lowering the inflammatory response is not efficacious to ameliorate PMS [19]. Further, a meta-analysis of 14 studies shows that treatment with either progesterone or progestogen showed no benefit in symptom reduction compared to placebo effects [19, see also, 20]. Rather, progestogens in second-generation pills (levonorgestrel or norethisterone) have been found to be regenerating PMS-type symptoms [3]. This suggests that the aetiology of PMS does not lie in differences in absolute levels of progesterone, but more likely in the cyclical variation of the hormone. Indeed, changes in hormone concentration, e.g. falling levels of progesterone in the days preceding the onset of the menses, are thought to carry more biological value that any given level at any given time [21].

One recent evolutionary hypothesis suggests that severe PMS might arise from the exacerbation of persistent infections [4, 5]. Doyle and Ewald proposed that the weakening of the immune response during the mid-luteal phase allows for the growth of pre-existing pathogen populations. Such infections might go unnoticed and persist, which is particularly expected in cases when natural selection favours low levels of virulence, such as sexually transmitted infections for which opportunities of transmission are low [22]. There is now some evidence that higher susceptibility to bacterial, viral and fungal infections in the low-inflammatory (mid-luteal)-phase of the cycle is associated with high levels of progesterone [12]. If cyclical immunosuppression allows for pathogen load to increase during the second (luteal) phase of the cycle, then the pre-menstrual drop in both progesterone and oestrogen levels provoking the onset of the inflammatory reaction eventually leading to menstruation will be heightened. This leads to exacerbated premenstrual symptoms.

In a pioneering study, Doyle and colleagues investigated the link between sexually transmitted infections and depression and other PMSx, in particular headache, pain and nausea [23]. The study was based on medical records for 500 regularly cycling women, of which 120 were diagnosed with PMS (24%). Infections investigated were both viruses (*Human papillomavirus*; HPV) and bacteria (*Chlamydia trachomatis*, *Neisseria gonorrheae*, *Gardnerella vaginalis*, *Candida albicans* and *Trichomonas vaginalis*). The main result was the association between *C. trachomatis* and two common PMSx: depression and pain. *T. vaginalis* was associated with headache while *G. vaginalis* was associated with nausea. Symptoms were not associated with lifestyle variables such as diet, smoking, alcohol consumption, drug use and exercise. However, given only the presence and not the timing of occurrence of symptoms was recorded, causality could not be inferred. In addition, the authors specify that the medical categorization of PMS had often been conflated with dysmenorrhoea (i.e. painful menstruation usually associated with abdominal cramps), which thus precludes distinguishing menstrual from premenstrual symptoms. Another recent study based on clinical files from 148 women evaluated retrospectively the link between, on the one hand, anti-bacterial treatment, cervical/stromal anti-inflammatory and antibiotic injections combined with intra-cervical cryotherapy, and on the other hand, self-rated PMS [24]. The study reports a positive effect of treatment, with a reduced mean score of severity of all 10 PMSx (depression, irritability, anxiety, fatigue, headache, oedema, breast tenderness, abdominal bloating, pelvic pain and dysmenorrhea). However, the study includes no control, and the conclusion relies on multiple non-parametric tests. Thus, the possibility that infection may exacerbate pre-menstrual symptoms warrants further investigation.

The main objective of this paper is to test the hypothesis that sexually transmitted infections (STIs) cause debilitating side-effects in the days leading to menstruation, a phase during which PMS typically occurs. Overall, this research is a first step towards determining under which conditions PMSx and STIs co-occur, and whether those symptoms might be reliable clinical indicators of undiagnosed conditions. To circumvent some of the limitations of previous studies, we collected data using the app *Clue*, a period-tracker that has 10 million of active users. Users were asked whether and when they had been tested for an infection, and the result of the test. We then paired those data with cycle data before and after the test for STIs. Cycle data include data on period length, cycle length and both pain (cramps, headache, tender-breasts) and emotions (sadness, happiness, sensitive emotions) as proxies for PMSx (Table 1). We ran analyses to answer the following two questions: (i) Prior to diagnosis, were PMSx more likely for women with an STI and unaware about it? (ii) Among individuals diagnosed positive for an infection, have symptoms decreased after treatment?

**Table 1.** Text given in the *Clue* app to describe the options for tracking emotion (happy, sensitive and sad) and pain (cramps, headache and tender breasts)

Tracking options	Description
*HAPPY*—Content and cheerful	Despite the dominant belief that the premenstrual phase and bad moods go hand in hand for everyone, some people experience positive moods in relation to their cycle. If you’re looking for patterns between your mood and your menstrual cycle, tracking both positive and negative mood is important.
*SENSITIVE*—Easily triggered	Some people report crying spells or feeling sensitive or tearful during the premenstrual phase.
*SAD*—Feeling a bit down	Some people report crying spells and feeling depressed, anxious, nervous or blue during the premenstrual phase. If you experience sadness that is regularly severe enough to interfere with your relationships or other aspects of day-to-day life, it may be a good time to talk to your doctor.
*CRAMPS—*Pain from cramping of the uterus, which may also be felt in the lower back and thighs	Cramps are common pain symptoms in the days before and during menstruation. Scientists suspect that cramps may be caused by an excess of prostaglandins, which are hormone-like substances that help the uterus contract to shed the uterine lining.
*HEADACHE—*A sustained ache in the head	Headaches are common premenstrual and menstrual symptoms. Prostaglandins, which are hormone-like substances that help the uterus contract to shed the uterine lining, have been proposed to play a role. Migraines are intense headaches that may be accompanied by pulsating pain, sensitivity to light and noise, nausea and/or vomiting. Migraines are classified as with or without aura. Many people with migraines report an association between menstruation and migraine incidence. People with migraines also report changes in migraine experience throughout their reproductive lifespan relating to first period, oral contraceptive use, pregnancy and/or menopause. Changing estrogen levels are believed to mediate these changes and taking extra estrogen (as hormonal birth control or hormonal replacement therapy) may make migraines worse.
*TENDER BREASTS—*Sore breasts due to water retention	Breast pain is a common premenstrual pain symptom. Pain is often diffuse and felt in both breasts. Increased water content due to changing hormone levels in the luteal phase is a likely factor.

The use of digital health technology for recruiting participants and data collection comes with both strengths and limitations. On the one hand, through its crowd-sourcing capability, the app enables one to recruit and reach a potentially large number of participants. Most importantly, the cycle data can be fine-grained (through daily entry) and longitudinal (over several cycles), which greatly enhances the potential for inferring causal rather than correlational effects. Finally, in the long-run, users can contribute to research while improving their understanding of their own health. On the other hand, however, data are indirect because information is self-reported, and levels of systemic inflammation are not known. Further, the tracking categories included in the app can be said to reproduce the cultural construction of PMS that is typical of Western settings (e.g. the US, Europe and Australia), where the occurrence of premenstrual changes is viewed as negative and pathological [16]. Thus, the study primarily informs on the role of STIs in modifying negative, rather than positive, premenstrual symptoms in Western populations. Ultimately, this study serves as a basis for reviewing digital health research capability and improving its tracking categories.

## METHODOLOGY

### The data

#### Study population: Clue users

Data were collected using a smart phone app, *Clue* [25], a period-tracker first released by BioWink GmbH in 2013. *Clue* enables users to collect daily data on menstrual bleeding, emotional well-being and behaviour and provide them with scientific information relating to menstrual health. This study focused on a sub-section of *Clue* users, recruiting only from users of the English version of the app and with a registered account with *Clue*. This is because users with an account have given their permission to use their cycle data for research.

#### Recruitment process

Before starting data collection, this study was granted ethical clearance from the Ethical Board of the School of Anthropology at the University of Oxford (reference SAME_C1A_16_048). The study was first announced and distributed through an e-mail based personal review of menstrual cycle data. This e-mail, referred to as ‘Cycle Review’ in the e-mail subject, is sent since June 2016 to registered users to situate the user’s data in relation to aggregated data from other users with similar characteristics. Starting in July 2016, *Clue* users could click on the link inviting them to participate to the study and could enter data after their informed consent was obtained. Based on Clue’s previous experience, it was agreed to include a link to the study in both July’s and August’s monthly e-mails and stop the data collection by the 31st of August 2016, a date after which it was expected that no more responses would be registered. However, given the relatively low sample size (<20) for the critical group (tested positive and off hormonal contraceptives) by the end of the data collection period, we decided to advertise the study on *Clue’*s blog and at an ‘influencer event’ taking place at *Clue* headquarters in September 2016. The data collection was finally stopped exactly 3 months after the first responses came in (by July the 19th), by October the 19th, after the additional advertizing push was over (see Supplementary Figure S1 for the distribution of survey responses across time). We used the *Typeform* platform to collect data and follow the data collection process.

#### Survey data

The survey included questions on key variables of interest, i.e. the occurrence and the date of the latest completed medical test for a sexually transmitted infection (STI), its result and treatment, if appropriate, along with confounding variables, i.e. age, the use of hormonal contraception (pill, patch, hormonal IUD, implant, injection), the use of vitamins and/or supplements, the regular uptake of any form of medication, the occurrence of unprotected sex as well as the reason for the test. Conflating variables are likely to influence the results in the following ways: first, whether women used hormonal contraceptives or not at the time of the STI test is critical because the hormonal contraceptives partly remove cyclical immunity and interfere with the immune system. Second, food supplements have been suggested to influence PMSx, and a few studies suggest that calcium and Vit B6 supplementation may be an effective treatment (reviewed in [3]). Third, the uptake of regular medication for treating chronic conditions such as autoimmune diseases, diabetes or endometriosis might influence hormonal levels and thus impact the relationship between STI and PMS in complex ways.

#### Menstrual cycle data

Survey data were combined with cycle data for registered *Clue* users who reported to have been tested for an infection in the past, for whom no multiple survey entries were detected, for whom cycle data before and after the cycle during which the test occurred were available and for whom cycles were within the normal range, as defined by the American College of Obstetricians and Gynaecologists [26], i.e. a cycle length ranging from 24 to 38 days and a period length of up to 8 days. For each survey participant, the cycle data belonging to two cycles were retrieved. The two cycles were chosen to be the closest full cycle before the test and closest full cycle after the results. For each day of the two cycles, data were extracted with regards to a number of symptoms both recorded in the app and known to be positively or negatively correlated with PMS: cramps, headache, tender breasts, happy, sad, sensitive emotions (see Table 1 for details given to participants for each tracking category). Each symptom category is presented on a screen with four options, which are not exclusive, and it is possible to select all symptoms together in the same day. For each day of each cycle, when no data have been entered for a particular symptom, the presence of a symptom was coded 0 only if other data had been entered that day, otherwise it was coded as missing data. In addition to symptomatic data, data on cycle length and period length for each cycle were added to the dataset.

### Statistical analysis

The analysis aims at answering the following two questions: (Q1) Does the presence of an undiagnosed infection exacerbate the occurrence of pre-menstrual symptoms? (Q2) Do PMS-like symptoms improve after diagnosis and treatment? All analyses were performed using the R software version 3.3.3. [27] and aggregate data are available on Figshare data repository [28]. Individuals data are not available to ensure the anonymity of participants.

#### (Q1) Does the presence of an undiagnosed infection exacerbate the occurrence of pre-menstrual symptoms?

This analysis investigates whether the probability of experiencing PMS-like symptoms is significantly different between STI+ and STI- individuals in the cycle just preceding the cycle during which participants took a medical test. A model was run for each of the pain and emotion symptoms available in the app and commonly associated with PMS (see Table 1 for a detailed description). Symptoms were kept as distinct response variables rather than combined through a principal component analysis because this method is usually not reliable in the case of dichotomous variables (Dr. Lunn, Oxford Statistics, pers. com). Explanatory variables were infection status (infected/not-infected), phase (early luteal/late luteal, detailed below), age (continuous), cycle length (continuous), regular use of medicine (yes/no), vitamins (yes/no) and hormonal contraceptive use (yes/no). Age and cycle length were not normally distributed thus these variables were log transformed prior to be entered in the models. Given that BMI is a poor measure of health [29] and there were many missing data for self-reported height and weight, we did not include this variable.

##### Mixed logistic regression with temporal autocorrelation

Symptoms can be recorded every day and were recorded on a binary scale (presence/absence), thus, we used a logistic regression routine. To account for the structure of the data (binary response variables, repeated data within individuals and within countries, temporal autocorrelation between the days of the cycle), we used a mixed logistic regression routine with varying intercepts for individual ID, and with a temporal autocorrelation structure for the residuals (cycle days nested within individuals). This was possible using the glmmPQL function of the *‘nlme’* package [30]. One advantage of multi-level analysis is that it can account for unbalanced designs [31], such as found in our data, thanks to the use of best unbiased linear prediction for estimating random effects. Intra-class correlations coefficients (% of between group variability in the observed variance) range from 0.86 to 0.92. The tables are displayed using the ‘*texreg’* package [32].

##### Determining the pre-menstrual phase

To test for the occurrence of a link between infection and the timing of the symptoms we included a variable *Cycle Phase* in each model to distinguish between symptoms reported in the early and in the late luteal phases. We restricted the data to the luteal phase (onset of menstruation—14 days) because: (i) premenstrual symptoms only occur in the luteal phase, (ii) for individuals diagnosed with an infection, it is not known when they were infected thus the closer the data are to the time of test the more likely diagnosed individuals were already infected, (iii) the luteal phase shows the least inter-individual variability compared to the follicular phase [33] making the comparison between women more meaningful and (iv) most of the data are collected during the menses, which distorts the signal towards the menstrual, follicular period.

It is not obvious when the pre-menstrual (i.e. the late luteal) phase should start. Pre-menstrual symptoms can start as early as 2 weeks before the menses according to the NHS [2], however in this paper we assume that symptoms occur as the result of falling progesterone levels and the subsequent activation of the immune response [34]. Thus, the relevant pre-menstrual phase should start when progesterone levels start falling, about 7 days before the menses [1]. To restrict the number of models performed, we assumed that the window within which the signal is likely to be the strongest is between 3 and 5 days before the menses (for 1 and 2 days, there might not be enough data and for 6 and 7 days, progesterone may not have fallen enough yet). We then created three dichotomous variables: *Cycle Phase 3d* [late luteal (3 days before the onset of the menses)/early luteal (other days)], *Cycle Phase 4d* [late luteal (4 days before the onset of the menses)/early luteal (other days)] and *Cycle Phase 5d* [late luteal (5 days before the onset of the menses)/early luteal (other days)].

##### Comparing models

To better understand the determinants of diversity in the experience of PMSx, the following model structure was used: Cycle Phase * Infection Status * Hormonal Contraceptives Use + log (Age) + log (Cycle length) + Supplements + Medication. For each PMSx, we ran and compared three models, each varying by how the late luteal phase was defined (either *3 days (Cycle Phase 3d), 4 days (Cycle Phase 4d) or 5 days (Cycle Phase 5d) before the menses)*. In each model, we included the interaction Cycle Phase * Infection Status to test for an effect of infection on the exacerbation of PMSx in the late luteal phase. We also included an interaction (*) with the uptake of hormonal contraceptives because the effect of infection on the experience of PMSx is predicted to be less visible among contraceptive users. This is because the magnitude of the rise and the fall of progesterone levels is less important among users of hormonal contraceptives [1].

The *glmmPQL* function uses penalized likelihood estimation, thus it is not possible to obtain an AIC and use standard model comparison techniques. We kept a frequentist approach and performed, for each model, a Wald χ2 test on all coefficients using the package ‘*aod*’ [35] to test coefficients jointly given their variance covariance matrix. For each model, we plotted a receiver operating characteristic curve (ROC) and computed a measure of the accuracy of the chosen model in predicting the data using the area under the curve (AUC). All measures of AUC were >94%. Estimates and confidence intervals were converted to odd-ratios using the inverse logit function *exp(x)/(1+exp(x))* in reporting the results. Effects that are independent of how the cycle phase is defined are considered robust.

#### (Q2) Do PMSx improve after diagnosis and treatment?

Based on the results of the first analysis (Q1), we investigated whether the relationship between infectious status and the occurrence of PMSx was reduced after treatment. Three groups of participants were compared: (i) participants tested and diagnosed positive, and who received treatment; (ii) participants tested and diagnosed positive, and who did not receive treatment and (iii) participants tested and diagnosed negative. To avoid including quadruple interactions and drawing on the results obtained for (Q1), we restricted the data to women who were not using hormonal contraception at the time of their test. We restricted the analysis to PMSx for which the relationship with infection status was found to be robust in (Q1). For each model, we plotted a receiver operating characteristic curve (ROC) and computed a measure of the accuracy of the model in predicting the data using the area under the curve (AUC). Estimates and confidence intervals were converted to odd-ratios using the inverse logit function *exp(x)/(1+exp(x))* in reporting the results.

## RESULTS

### Descriptive statistics

A total of 34 511 individuals started the survey, of which 30 605 gave their informed consent. Among those, 33% (10 054) reported to have ever been tested for an infection. The infections recorded were HPV (34%), *C. trachomatis* (33%), *Herpes simplex virus* (24%), *N. gonorrhea* (4%), *T. vaginalis* (4%), *C. albicans* (3%), syphilis (*Treponema pallidum*) (<1%), hepatitis (<1%) and others (7%). From the pool of users who had ever been tested for an infection, we restricted the sample to users with the relevant data, i.e. age and complete cycles before and after the cycle during which testing occurred, and with cycles within the normal range, as defined by the American College of Obstetricians and Gynaecologists [26], i.e. a cycle length ranging from 24 to 38 days and a period length of up to 8 days. Further, we restricted our analysis to individuals aged 18–45 years because teenagers and pre-menopausal women often have irregular cycles and low hormonal production [for review see, 36]. In the final sample used for analysis (*N* = 865), which includes both hormonal contraceptive users (*N* = 241) and non-users (*N* = 624), the median age of users at the time of the survey was 26 years, the median cycle length was 28 days and the median period length was 4 days (Table 2). In the final sample, 6.7% (*N* = 58) of individuals tested positive, of which 60% were prescribed a treatment (*N* = 35), which was completed by 96% of individuals. Most users were from English speaking countries (the US: 59%; the UK: 11.9%; Canada: 8.2%; Australia, 5.6%).

**Table 2.** Descriptive statistics

	Infection status: Positive			Infection status: Negative			All
	HC users	HC non-users	All	HC users	HC non-users	All	
*N*	18	40	58	223	584	807	865
Median age	26	27	26	24	26	25	26
Median cycle length	28	30	29	28	29	28	28
Median period length	4	4	5	5	4	4	4
Vitamins and/or Supplements (*N*)	8	18	26	97	327	424	450
Medicine (*N*)	4	14	18	74	211	285	303
Unprotected Sex (*N*)	6	8	14	56	132	188	202

N: Sample size; HC: Hormonal contraceptives.

### Inferential statistics

#### Do women with an undiagnosed sexually transmitted infection experience more pre-menstrual symptoms (Q1)?

**Figure 1.**
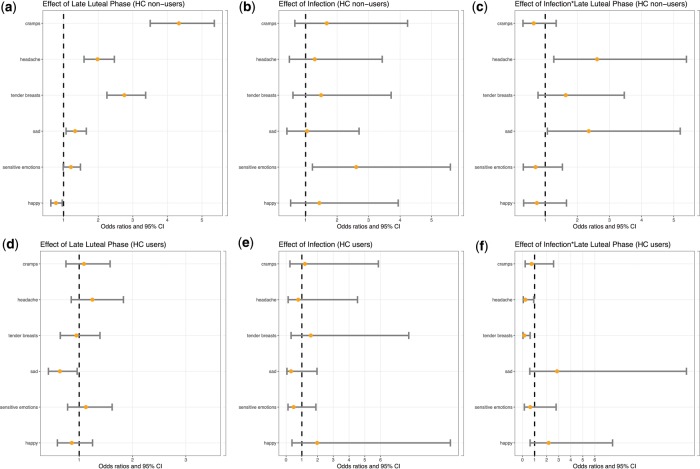
Predicted odds (circles) and 95% confidence intervals (horizontal bars) of experiencing pain and emotions during the menstrual cycle as a function of hormonal contraceptive use, the phase of the menstrual cycle and the presence of a sexually transmitted infection (STI). Among non-users of hormonal contraceptives (top panel, *N *= 624), (**a**) the odds of experiencing symptoms are higher (e.g. cramps, headache, tender breasts and sadness) or lower (i.e. happy) in the late luteal phase (−3 days to the onset of menses), as compared to the early luteal phase (dashed line); (**b**) the presence of an infection is associated with an increased odds of experiencing sensitive emotions throughout the luteal phase as compared with the absence of a STI (dashed line); (**c**) the odds of experiencing a headache and sadness in the late luteal phase is higher for individuals with a STI (*N *= 40) as compared with individuals without a STI (dashed line). Among users of hormonal contraceptives (bottom panel, *N *= 241), (**d**) the odds of experiencing sadness is lower in the late luteal phase as compared with the early luteal phase; (**e**) the odds of experiencing any of the symptoms is not modified by the presence of a STI, as compared with the absence of a STI (dashed line); (**f**) the presence of a STI decreases the odds of experiencing tender breasts in the late luteal phase

This analysis is based on a between-women design and uses data recorded during the cycle prior to the one during which women undertook a STI test. We used a multi-level logistic regression routine to investigate the role of infectious status for explaining variation in the experience of PMSx both during the late luteal phase and during the wider luteal phase. The results depend on the symptom investigated and on whether or not women are using hormonal contraception at the time of the test (Fig. 1).

*(i) Women off hormonal contraception (N = 624).* We found robust evidence that the presence of an undiagnosed infection is associated with the exacerbation of headaches in the days before the onset of the menses, independently of how the pre-menstrual phase is defined. STIs are also associated with an increase in the overall experience of sensitive emotions.

*Physical symptoms*. Cramps, headache and tender breasts appear to be valid PMSx as they are more likely to be experienced in the late luteal phase as compared with the rest of the luteal phase *(**Table 3**)*. *Cramps:* STI+ individuals are ca. 2 times (OR = 2.41, 95CI [1.08; 5.38]) more likely to experience cramps during the 5 days preceding the onset of their periods as compared with non-infected women. This effect is not observed when the late luteal phase is defined as 3 or 4 days before the menses, however. *Headache*: STI+ individuals are ca. 2 times more likely to experience headache during the last 5 days of their cycle compared with the rest of the luteal phase (OR = 2.36, 95CI [1.19; 4.69]), and this effect is also observed when the late luteal phase is defined as 3 or 4 days before the menses. *Tender breasts*: the presence of infection does not exacerbate this physical premenstrual symptom (OR = 1.95, 95CI [0.90; 4.19]).

**Table 3.** STI status and the experience of pain during the luteal phase of the menstrual cycle

	Cramps (−3d)	Cramps (−4d)	Cramps (−5d)	Headache (−3d)	Headache (−4d)	Headache (−5d)	Tend.Br. (−3d)	Tend.Br. (−4d)	Tend.Br. (−5d)
Intercept	−13.43***	−13.26***	−13.26***	1.02	0.78	0.69	−2.75	−2.99	−3.19
	(3.51)	(3.46)	(3.45)	(3.84)	(3.82)	(3.83)	(3.55)	(3.57)	(3.58)
Inf (1)	0.51	0.37	−0.24	0.25	0.32	0.23	0.40	0.61	0.28
	(0.48)	(0.48)	(0.52)	(0.50)	(0.50)	(0.51)	(0.47)	(0.47)	(0.50)
Phase (LL)	1.47***	1.29***	1.14***	0.68***	0.54***	0.53***	1.01***	1.18***	1.30***
	**(0.11)**	**(0.11)**	**(0.11)**	**(0.11)**	**(0.11)**	**(0.11)**	**(0.10)**	**(0.10)**	**(0.11)**
Horm (Yes)	−0.13	−0.22	−0.27	−0.30	−0.23	−0.19	−0.51*	−0.41	−0.24
	(0.24)	(0.24)	(0.24)	(0.25)	(0.25)	(0.26)	**(0.24)**	(0.25)	(0.25)
Log (Age)	0.13	0.15	0.15	−0.60	−0.55	−0.54	0.67	0.69	0.67
	(0.48)	(0.47)	(0.47)	(0.51)	(0.51)	(0.51)	(0.47)	(0.48)	(0.48)
Log (Clength)	2.74**	2.68**	2.68**	−1.00	−0.98	−0.96	−0.72	−0.73	−0.70
	**(0.88)**	**(0.87)**	**(0.87)**	(0.97)	(0.97)	(0.97)	(0.90)	(0.90)	(0.91)
Vit (Yes)	0.23	0.23	0.22	0.39	0.38	0.38	0.02	0.01	0.02
	(0.19)	(0.19)	(0.19)	(0.21)	(0.21)	(0.21)	(0.19)	(0.20)	(0.20)
Med (Yes)	0.26	0.29	0.29	0.70**	0.70***	0.70**	0.07	0.07	0.07
	(0.20)	(0.19)	(0.19)	**(0.21)**	**(0.21)**	**(0.21)**	(0.20)	(0.20)	(0.20)
Inf (1)* Phase(LL)	−0.45	−0.14	0.88*	0.96**	0.75*	0.86*	0.49	0.06	0.67
	(0.38)	(0.37)	**(0.41)**	**(0.37)**	**(0.35)**	**(0.35)**	(0.38)	(0.35)	(0.39)
Inf (1)* Horm (Y)	0.18	−0.05	0.69	−0.26	−0.53	−0.59	0.45	0.34	0.74
	(0.81)	(0.86)	(0.89)	(0.90)	(0.91)	(0.93)	(0.82)	(0.83)	(0.87)
Phase( LL)* Horm(Y)	0.09	0.20	0.26	0.22	0.00	−0.07	−0.05	−0.23	−0.47*
	(0.19)	(0.19)	(0.19)	(0.20)	(0.19)	(0.19)	(0.20)	(0.19)	**(0.19)**
Inf (1)*Phase(LL)* Horm(Y)	−0.29	0.09	−1.14	−1.42*	−0.70	−0.55	−1.89**	−1.42*	−1.96**
	(0.63)	(0.66)	(0.69)	**(0.69)**	(0.63)	(0.62)	**(0.73)**	**(0.71)**	**(0.71)**

*** *P* < 0.001,

** *P* < 0.01,

* *P* < 0.05.

Standard errors are provided in brackets. *Inf*: Infection status (0: diagnosed negative, 1: diagnosed positive); *Phase* (EL: early luteal; LL: late luteal (3–5 days before the onset of the menses)); *Clength*: cycle length; *Horm* (whether individuals use hormonal contraceptives at the time of test (Yes/No)); *Vit* (whether individuals take vitamins and/or supplements at the time of test (Yes/No)); *Med* (whether individuals take regular medication at the time of test (Yes/No)); * denotes an interaction. Bold depicts significance; *ICC_id*: intra-class correlation at the level of women, that is, the amount of variance left to be explained at the individual level; *Phi* is the coefficient of temporal autocorrelation between days within individuals; a Wald test is used to test the joint significance of all variables included in each full model.

*Psychological symptoms.* Sad, sensitive emotions and happiness appear to be valid PMSx as they are more likely to be experienced during the late luteal phase as compared with the rest of the luteal phase *(**Table 4**)*. *Sensitive emotions*: STI+ individuals are >2 times more likely to experience sensitive emotions (OR = 2.61, 95CI [1.21; 5.60]), independently of the phase of the menstrual cycle. *Sad*: STI+ individuals are >2 times more likely to experience sadness in the 3 days preceding the menses (OR = 2.36, 95CI [1.07, 5.17]), but this effect is not robust to how the late luteal phase is defined. *Happy*: the presence of an infection does not further decrease happiness in the late luteal phase (OR = 0.74, 95CI [0.33; 1.65]).

**Table 4.** STI status and the experience of emotions during the luteal phase of the menstrual cycle

	Sad (−3d)	Sad (−4d)	Sad (−5d)	Sensitive (−3d)	Sensitive (−4d)	Sensitive (−5d)	Happy (−3d)	Happy (−4d)	Happy (−5d)
Intercept	−7.01	−7.03	−7.02	−7.14*	−7.22*	−7.31*	−2.30	−2.17	−2.06
	(3.60)	(3.59)	(3.59)	**(2.99)**	**(2.99)**	**(3.00)**	(3.86)	(3.86)	(3.87)
Inf (1)	0.04	0.32	0.39	0.96*	0.99*	1.11**	0.36	0.16	0.12
	(0.48)	(0.48)	(0.48)	**(0.39)**	**(0.40)**	**(0.40)**	(0.51)	(0.52)	(0.52)
Phase (LL)	0.29**	0.35***	0.22*	0.19	0.35***	0.45***	−0.25*	−0.43***	−0.47***
	**(0.11)**	**(0.11)**	**(0.10)**	(0.10)	**(0.10)**	**(0.10)**	**(0.11)**	**(0.10)**	**(0.10)**
Horm (Yes)	−0.10	−0.10	−0.20	−0.19	−0.13	−0.07	−0.00	−0.04	−0.10
	(0.23)	(0.24)	(0.24)	(0.20)	(0.20)	(0.21)	(0.25)	(0.25)	(0.25)
Log (Age)	0.27	0.28	0.29	0.13	0.13	0.12	−0.31	−0.32	−0.32
	(0.49)	(0.49)	(0.49)	(0.41)	(0.41)	(0.41)	(0.53)	(0.53)	(0.53)
Log (Clength)	0.71	0.70	0.70	1.19	1.20	1.21	0.08	0.07	0.05
	(0.91)	(0.91)	(0.91)	(0.76)	(0.76)	(0.76)	(0.98)	(0.98)	(0.98)
Vit (Yes)	0.14	0.14	0.14	0.09	0.09	0.10	0.18	0.18	0.18
	(0.20)	(0.20)	(0.20)	(0.17)	(0.17)	(0.17)	(0.21)	(0.22)	(0.22)
Med (Yes)	0.13	0.13	0.13	0.14	0.14	0.14	0.52*	0.53*	0.53*
	(0.21)	(0.20)	(0.20)	(0.17)	(0.17)	(0.17)	**(0.22)**	**(0.22)**	**(0.22)**
Inf (1) * Phase (LL)	0.86*	0.20	0.05	−0.36	−0.34	−0.54	−0.30	0.29	0.32
	**(0.40)**	(0.39)	(0.39)	(0.40)	(0.37)	(0.35)	(0.41)	(0.38)	(0.36)
Inf (1)* Horm (Y)	−1.15	−1.66	−1.24	−0.74	−0.68	−0.65	0.68	0.72	0.51
	(0.93)	(0.96)	(0.93)	(0.70)	(0.72)	(0.73)	(0.85)	(0.86)	(0.87)
Phase (LL)* Horm (Y)	−0.45*	−0.33	−0.06	0.12	−0.01	−0.11	−0.15	−0.04	0.12
	**(0.21)**	(0.19)	(0.18)	(0.19)	(0.17)	(0.17)	(0.19)	(0.18)	(0.17)
Inf (1)* Phase (LL)* Horm (Y)	1.05	1.66*	0.86	−0.44	−0.53	−0.55	0.77	0.53	0.85
	(0.80)	**(0.78)**	(0.72)	(0.75)	(0.69)	(0.67)	(0.63)	(0.58)	(0.57)

*** *P* < 0.001,

** *P* < 0.01,

* *P* < 0.05.

Standard errors are provided in brackets. *Inf*: Infection status (0: diagnosed negative, 1: diagnosed positive); *Phase* (EL: early luteal; LL: late luteal (3–5 days before the onset of the menses)); *Clength*: cycle length; *Horm* (whether individuals use hormonal contraceptives at the time of test (Yes/No)); *Vit* (whether individuals take vitamins and/or supplements at the time of test (Yes/No)); *Med* (whether individuals take regular medication at the time of test (Yes/No)); ‘*’ denotes an interaction. Bold depicts significance; *ICC_id*: intra-class correlation at the level of women, that is, the amount of variance left to be explained at the individual level; *Phi* is the coefficient of temporal autocorrelation between days within individuals; a Wald test is used to test the joint significance of all variables included in each full model.

*(ii) Women on hormonal contraception (N = 241).* A link between infection and the exacerbation of pain and emotions in the late luteal phase is never observed among contraceptive users. Using hormonal contraceptives mostly relates to the experience of tender breasts, and to a lesser extent, sadness.

*Physical symptoms. Cramps:* Hormonal contraceptive users do not experience less cramps as compared with non-users (OR = 0.76, 95CI [0.48; 1.22]), even in the presence of an infection (OR = 1.99, 95CI [0.35; 11.40]), or during the late luteal phase (OR = 1.30, 95CI [0.89; 1.88]) *(**Table 3**)*. *Headaches*: Hormonal contraceptive users do not experience less headaches as compared with non-users (OR = 0.79, 95CI [0.49; 1.30]), even in the presence of an infection (OR = 0.59, 95CI [0.10; 3.50]), or during the late luteal phase (OR = 1, 95CI [0.69; 1.45]). *Tender Breasts*: Users of hormonal contraception experience less symptoms in the last 3 days of their cycle (OR = 0.60, 95CI [0.37, 0.96]), but the result is not robust to how the pre-menstrual phase is defined.

*Psychological symptoms. Sad*: Users of hormonal contraception and diagnosed with an infection experience less sadness in the last 3 days of their cycle (OR = 0.64, 95CI [0.42; 0.96]), but the effect is not robust to how the pre-menstrual phase is defined *(**Table 4**)*. *Sensitive emotions*: Hormonal contraceptive users do not experience less sensitive emotions as compared with non-users (OR = 0.83, 95CI [0.56, 1.22]), even in the presence of an infection (OR = 0.50, 95CI [0.12, 2.07]), or during the late luteal phase (OR = 0.99, 95CI [0.71, 1.38]). Happy: Hormonal contraceptive users do not experience more happiness as compared with non-users (OR = 1, 95CI [0.64; 1.57]), in the presence of an infection (OR = 1.97, 95CI [0.37; 10.44]), or during the late luteal phase (OR = 0.86, 95CI [0.59; 1.25]).

#### Do women diagnosed with a sexually transmitted infection experience less symptoms after treatment (Q2)?

We investigated whether PMSx are reduced after the diagnosis and/or treatment of an STI (***Table 5)***. We restricted the analysis to women off hormonal contraceptives because we did not observe an exacerbation of PMSx among users of hormonal contraception. We also restricted our analyses to the two symptoms found to be the most influenced by the presence of an infection: headache and sensitive emotions. Three groups of women were compared: (i) women diagnosed negative for a STI (*N *= 585), (ii) women diagnosed positive for a STI but not receiving treatment (*N *= 14), (iii) women diagnosed positive for a STI and receiving treatment (*N *= 25). Among individuals diagnosed STI-, diagnosis alone is not sufficient to reduce headache (OR = 1.06, 95CI [0.84, 1.34]) or sensitive emotions (OR = 1.20, 95CI [0.96, 1.48]). Among individuals diagnosed STI+, headache does not decrease following treatment (OR = 1.23, 95CI [0.38, 3.99]) but, counter-intuitively, reduces in the absence of a medical prescription for the STI (OR = 0.00, 95CI [0.001, 0.12]). Conversely, the odds of experiencing sensitive emotions reduce in the cycle following both diagnosis and treatment (OR = 0.36, 95CI [0.14, 0.88]) while no changes are observed for STI+ individuals who did not receive treatment (OR = 1.99, 95CI [0.57, 6.99]).

**Table 5.** The effects of STI diagnosis and treatment on the experience of headache and emotions in the luteal phase

	Headache (−3d)	Headache (−4d)	Headache (−5d)	Sensitive (−3d)	Sensitive (−4d)	Sensitive (−5d)
Intercept	−13.24***	−13.15***	−13.34***	−8.38***	−8.38***	−8.40***
	**(2.59)**	**(2.58)**	**(2.59)**	**(2.09)**	**(2.10)**	**(2.10)**
Group(P/NoT)	−1.70	−1.46	−1.56	0.70	0.57	0.64
	(0.96)	(0.95)	(0.96)	(0.53)	(0.55)	(0.55)
Group(P/T)	0.64	0.67	0.55	1.06**	1.15**	1.31**
	(0.49)	(0.49)	(0.51)	**(0.40)**	**(0.41)**	**(0.42)**
Time (After)	0.10	0.09	0.06	0.10	0.16	0.18
	(0.11)	(0.12)	(0.12)	(0.09)	(0.10)	(0.11)
Phase (LL)	0.63***	0.50***	0.48***	0.22*	0.38***	0.48***
	**(0.12)**	**(0.12)**	**(0.12)**	**(0.11)**	**(0.11)**	**(0.10)**
Log (Age)	0.23	0.25	0.24	0.42	0.40	0.39
	(0.43)	(0.43)	(0.43)	(0.34)	(0.34)	(0.34)
Log (Clength)	2.53***	2.49***	2.54***	1.32**	1.31**	1.31**
	**(0.60)**	**(0.60)**	**(0.60)**	**(0.49)**	**(0.49)**	**(0.49)**
Vit (YES)	0.19	0.18	0.17	0.01	0.02	0.02
	(0.18)	(0.18)	(0.18)	(0.14)	(0.14)	(0.14)
Med (YES)	0.59**	0.59**	0.59**	0.14	0.14	0.14
	**(0.18)**	**(0.18)**	**(0.19)**	(0.15)	(0.15)	(0.15)
Group (P/NoT)* Time (After)	1.45	1.40	2.13*	−0.35	−0.19	−0.69
	(1.01)	(1.02)	**(1.06)**	(0.57)	(0.59)	(0.64)
Group (P/T)* Time (After)	−0.25	−0.18	−0.17	−0.59	−0.79	−1.03*
	(0.36)	(0.37)	(0.40)	(0.40)	(0.42)	**(0.46)**
Group (P/NoT)* Phase (LL)	3.13**	2.67**	2.76**	0.11	0.36	0.25
	**(0.98)**	**(0.95)**	**(0.96)**	(0.60)	(0.57)	(0.57)
Group (P/T)* Phase (LL)	0.16	0.10	0.32	−0.57	−0.72	−0.96*
	(0.47)	(0.44)	(0.42)	(0.55)	(0.50)	**(0.48)**
Time (After)* Phase (LL)	−0.12	−0.06	−0.00	−0.02	−0.16	−0.19
	(0.17)	(0.16)	(0.16)	(0.15)	(0.15)	(0.14)
Group (P/NoT)* Time (After)* Phase (LL)	−3.94**	−3.68**	−5.02***	−1.78	−1.69	−0.24
	**(1.36)**	**(1.34)**	**(1.48)**	(1.17)	(1.02)	(0.87)
Group (P/T)*Time (After)*Phase (LL)	0.03	−0.17	−0.21	0.13	0.61	1.06
	(0.66)	(0.62)	(0.60)	(0.76)	(0.70)	(0.68)

*** *P* < 0.001,

** *P* < 0.01,

* *P* < 0.05.

Standard errors are provided in brackets. *Group*: among individuals tested for an infection, whether the result was negative (reference category), positive leading to treatment (P/T) or positive not leading to treatment (P/NoT); *Time*: whether the data are taken from the cycle before the test of after the results (Before/After); *Phase* (EL: early luteal; LL: late luteal (3–5 days before the onset of the menses)); *Clength*: cycle length; *Vit:* whether individuals take vitamins and/or supplements at the time of test (Yes/No)); *Med* (whether or not individuals take regular medication at the time of test (Yes/No)); * denotes an interaction. Bold depicts significance. Only non-users of hormonal contraception are considered in this analysis.

## DISCUSSION

Why women experience negative premenstrual symptoms has recently been linked to inflammation [13, 14, 24], and it has been suggested that PMS is best conceptualized as an ‘inflammatory disease’ [24]. While the proximate causes of premenstrual inflammation might be well understood, i.e. falling levels of progesterone near the onset of the menses activate an inflammatory response, the ultimate reasons why there is variation in levels of inflammation between and within women has received little attention (but see [23, 24]). In this paper, we investigate the possibility that negative premenstrual symptoms are exacerbated by the presence of a sexually transmitted infection using digital health data from *Clue*, a period-tracker app for smart phones. This hypothesis, put forward by Doyle and Ewald [34], posits that a pre-existing pathogen load would grow in the mid-luteal phase as a result of the anti-inflammatory environment promoted by high progesterone levels. This increased pathogen load would lead to the exacerbation of inflammatory symptoms pre-menstrually, when progesterone levels are falling. The hypothesis focuses on STIs because they are good candidates for chronic diseases: according to the virulence-transmission trade-off hypothesis [22], persistence within host is more likely to be favoured by natural selection when opportunities for transmission are infrequent, as is the case for STIs [23]. We find that, as predicted, some premenstrual symptoms are exacerbated by the presence of a STI among individuals who do not use hormonal contraceptives. The results have implications for both understanding the role of infection in modifying the premenstrual experience and using digital health for understanding female’s health.

The results show that in the cycle prior to undergoing a test for a sexually transmitted infection, individuals then diagnosed with one or several infections are at increased odds of experiencing headache, and a slightly longer period of cramps, before their menses. Cramps and headache are common inflammatory symptoms [37] and thus the results are in line with the idea that cyclical immunity enables STIs to grow and cause a heightened inflammatory reaction near the menses [23]. In addition, we found that among STI positive individuals, the odds of experiencing headache was reduced in the cycle just following diagnosis, but only in the group of women who did not receive a medical prescription. In this group, individuals were nearly all infected with *human papillomavirus* (85%), of which most strains clear up spontaneously without treatment [38]. It is also not excluded that headaches were less reported because individuals were relieved to learn that they were infected with HPV rather than with another incurable pathogen. By contrast, in the group receiving a medical prescription for the infection, individuals were mostly infected with *C.trachomatis* and *Herpes simplex virus* and symptoms did not improve in the cycle following treatment. It is likely that the infection takes longer than one cycle to clear up, but then information on the likelihood of reinfection in subsequent cycles would be needed to investigate this scenario. The absence of improvement could also partly be due to existing or rapid evolution of resistance to antibiotics in the case of Chlamydia, or a low efficacy of antiviral in the case of Herpes, and/or a counterproductive effect of anti-inflammatory medicine, preventing the normal body defence to fight off the infection. Given different pathogens present different prognosis (e.g. *C.trachomatis* is curable, *Herpes simplex virus* is not), further research looking into infection-specific effects over the long-term and using a larger sample size is warranted to evaluate the impact of treatment on negative premenstrual changes.

The results show that prior to diagnosis, the presence of an infection is associated with both an exacerbation of sensitive emotions throughout the luteal phase and sadness in the last 3 days of the cycle. Those results are in line with previous studies documenting a link between depression and *C. trachomatis* [23], or depression and infections more generally [39]. However, the link between ‘*sensitive emotions*’ and infection is not stronger in the late luteal phase, as compared with the early luteal phase, which might indicate that an increased experience of sensitive emotions among STI positive women is primarily driven by non-cyclical effects. For instance, individuals with an undiagnosed infection might be aware of the possibility of being infected, leading to low mood throughout the cycle. Indeed, in our sample, 60% of STI positive individuals asked for a test compared to a routine exam. It is also possible that the relationship is the other way around, as it has been shown that individuals experiencing chronic psychological stress are more susceptible to infectious diseases [39]. Finally, the question of whether the association between STIs and the experience of sensitive emotions is similar across various infectious diseases cannot be answered with our data due to low sample size per type of infectious agent. We cannot exclude the possibility that the relationship between sensitive emotions and STI is driven by certain infections due to particular immune-to-brain signalling pathways [40, 41].

A relationship between, on the one hand, psychological and physical negative symptoms, and on the other hand, STIs, is generally not observed among users of hormonal contraception. This was expected as progesterone levels do not cycle as much following the action of hormonal contraceptives [1]. Among contraceptive users, an undiagnosed pathogen load is thus not predicted to grow during the luteal phase of the cycle, and no or little exacerbation of PMS symptoms is anticipated. Although the use of hormonal methods of birth control is, in general, expected to remove immune cyclicity, its effect on the immune system is likely to be method-specific [42]. Given that information on the type of hormonal contraceptives used is not available in this study, it cannot be excluded that the absence of a relationship between the presence of a STI and a negative premenstrual experience among hormonal contraceptive users is characteristic of some methods but not of others. In this sample, the main robust correlate of hormonal contraceptive use is the reduced experience of breast tenderness pre-menstrually, which replicates previous findings [41, 43, but see, 44]. In naturally cycling women, ‘*tender breasts*’ is caused by increases in oestrogen and progesterone in the luteal phase, which cause an enlargement of milk ducts and milk glands, respectively [45]. In contraceptive users, the peaks of oestrogen (just before mid-cycle) and progesterone (around 21 days) of the cycle do not occur, preventing the occurrence of breasts tenderness.

### Limitations

This study makes use of digital health data, which presents both advantages and limitations. On the one hand, it is possible to access daily cycle data on a potentially large sample of individuals. On the other hand, the data are self-reported, data entry is biased towards specific days of the cycle, and the sample size reduces considerably once the questionnaire is paired with retrospective cycle data (the sample was reduced to 10% of its original size). In addition, the sample is largely biased towards WEIRD women, i.e. individuals from western, educated, industrialized, rich and democratic countries [48], thus it is not possible to generalise our findings to non-WEIRD populations. Note, however, that *Clue* cannot be said to be biased toward heterosexual individuals as, based on a recent representative and large survey of *Clue* users, 18% of individuals identify as bisexual/pansexual, lesbian/gay/homosexual, or queer. Another limitation of our study is that only two cycles were used, the one before the medical test and the one after results, which is not sufficient given that significant hormonal variation is found across cycles within women [49]. However, limiting the analysis to those cycles was necessary given that retrospective data do not inform on the exact timing of infection, and whether or not individuals were re-infected after diagnosis. Long-term prospective studies monitoring the clearing up of an infection across several cycles is the next step for investigating the link between infections and PMS. Further, it is not known whether participants were tested for all possible STIs. Thus, it is possible that some individuals received a negative diagnosis for some STIs while being infected with other STIs without knowing it. If this is the case with our data, then our ability to observe a difference between STI positive and STI negative individuals is likely to have been weakened, and thus the impact of STIs on negative premenstrual symptoms might actually be stronger than that observed. Finally, at the time of the study, the PMSx recorded in the app could be seen to reproduce the ‘hetero-patriarchal discourse’ framing the lived experience of the pre-menstrual phase as a negative one [16]. Out of the six options for tracking PMSx, only the ‘happy’ option allowed individuals to record a positive experience. This bias limits our ability to investigate the impact of infections on positive premenstrual symptoms, for instance energy and creativity [15, 46]. To provide a more complete picture of the premenstrual experience, apps should include more options for tracking positive pre-menstrual feelings and behaviours [15, 47]. In this line, the authors have started enquiring *Clue* users about their positive symptoms [15].

This study investigated the evolutionary hypothesis that a negative premenstrual experience can be exacerbated by the presence of a STI using digital health data from Western populations. The analysis suggests that the presence of STIs do increase the odds of experiencing headache, sadness and cramps pre-menstrually, and sensitive emotions throughout the luteal phase. However, further prospective work studying one pathogen at a time and using immunological data over several cycles is warranted to further our understanding of the link between the pre-menstrual experience and the occurrence of infections.

## Supplementary data

Supplementary data is available at *EMPH* online.

## FUNDING

The study is funded by a Economic and Social Research Council (UK) - Impact Acceleration Account Knowledge Exchange Fellowship ES/M500355/1.

**Conflict of interest**: None declared.

## Supplementary Material

Supplementary Data

## References

[1] Dalton K. Once a Month. 5th edn Alameda: Hunter Ho, 1999.

[2] Choices N. Premenstrual Syndrome (PMS). 2015 https://www.nhs.uk/conditions/pre-menstrual-syndrome/ (16 July 2018, date last accessed).

[3] Green LJ, O'Brien PMS, Panay N, Craig M on behalf of the Royal College of Obstetricians and Gynaecologists. Management of premenstrual syndrome. BJOG 2017; 124:e73–105.27900828

[4] Lanza di Scalea T, Pearlstein T. Premenstrual dysphoric disorder. Psychiatr Clin North Am 2017; 40:201–16. 28477648 10.1016/j.psc.2017.01.002

[5] Lopez LM, Kaptein AA, Helmerhorst FM. Oral contraceptives containing drospirenone for premenstrual syndrome. Cochrane Database Syst Rev 2009; (1):CD006586. 10.1002/14651858.CD006586.pub3 19370644

[6] Blom JMC, Ottaviani E. Immune-neuroendocrine interactions: evolution, ecology, and susceptibility to illness. Med Sci Monit Basic Res 2017; 23:362–7. 29142191 10.12659/MSMBR.907637 PMC5701458

[7] Teklenburg G, Salker M, Molokhia M et al Natural selection of human embryos: decidualizing endometrial stromal cells serve as sensors of embryo quality upon implantation. PLoS One 2010; 5:e10258. 20422011 10.1371/journal.pone.0010258 PMC2858159

[8] Salker M, Teklenburg G, Molokhia M et al Natural selection of human embryos: impaired decidualization of endometrium disables embryo-maternal interactions and causes recurrent pregnancy loss. PLoS One 2010; 5:e10287. 20422017 10.1371/journal.pone.0010287 PMC2858209

[9] Macklon NS, Brosens JJ. The human endometrium as a sensor of embryo quality. Biol Reprod 2014; 91:1–8. 10.1095/biolreprod.114.122846 25187529

[10] Gellersen B, Brosens JJ. Cyclic decidualization of the human endometrium in reproductive health and failure. Endocr Rev 2014; 35:851–905. 25141152 10.1210/er.2014-1045

[11] Segner H, Verburg-van Kemenade BML, Chadzinska M. The immunomodulatory role of the hypothalamus-pituitary-gonad axis: proximate mechanism for reproduction-immune trade offs? Dev Comp Immunol 2017; 66:43–60. 27404794 10.1016/j.dci.2016.07.004

[12] Alvergne A, Högqvist Tabor V. Is female health cyclical? Evolutionary perspectives on menstruation. Trends Ecol Evol 2018; 33:399–414. 29778270 10.1016/j.tree.2018.03.006

[13] Bertone-Johnson ER, Ronnenberg a. G, Houghton SC et al Association of inflammation markers with menstrual symptom severity and premenstrual syndrome in young women. Hum Reprod 2014; 29:1987–94. 25035435 10.1093/humrep/deu170

[14] Gold EB, Wells C, Rasor MO. The association of inflammation with premenstrual symptoms. J Women’s Heal 2016; 25:865–74. 10.1089/jwh.2015.5529 PMC5311461 27135720

[15] Clue AA. *Positive Symptoms of PMS*, 2016 https://helloclue.com/articles/cycle-a-z/positive-symptoms-pms. (17 July 2018, date last accessed).

[16] Ussher JM, Perz J. PMS as a gendered illness linked to the construction and relational experience of hetero-femininity. Sex Roles 2013; 68:132–50.

[17] Dantzer R, Capuron L. Inflammation-Associated Depression: Evidence, Mechanisms and Implications. New York: Springer, 2017.

[18] Myers JS. Proinflammatory cytokines and sickness behavior: implications for depression and cancer-related symptoms. Oncol Nurs Forum 2008; 35:802–7. 18765326 10.1188/08.ONF.802-807

[19] Wyatt K, Dimmock P, Jones P et al Efficacy of progesterone and progestogens in management of premenstrual syndrome: systematic review. BMJ 2001; 323:776–776. 11588078 10.1136/bmj.323.7316.776 PMC57352

[20] Ford O, Lethaby A, Roberts H et al Progesterone for premenstrual syndrome. Cochrane Database Syst Rev 2012; (3):CD003415. 10.1002/14651858.CD003415.pub4 PMC7154383 22419287

[21] Kol S, Homburg R. Change, change, change: hormonal actions depend on changes in blood levels. Hum Reprod 2008; 23:1004–6. 18326517 10.1093/humrep/den061

[22] Ewald PW. Evolution of Infectious Disease. New York: Oxford University Press, 1994.

[23] Doyle C, Swain WA, Swain Ewald HA et al Sexually transmitted pathogens, depression, and other manifestations associated with premenstrual syndrome. Hum Nat 2015; 26:277–91. 26272230 10.1007/s12110-015-9238-3

[24] Lolas-Talhami J, Lafaja-Mazuecos J, Ferrández-Sempere D. Is premenstrual syndrome a uterine inflammatory disease? Retrospective evaluation of an etiologic approach. Open J Obstet Gynecol 2015; 05:5: 305–12.

[25] Clue. 2018 https://helloclue.com/app.html (18 July 2018, date last accessed).

[26] ACOG. Abnormal Uterine Bleeding. American College of Obstetricians and Gynecologists. 2017 https://www.acog.org/Patients/FAQs/Abnormal-Uterine-Bleeding (17 July 2018, date last accessed).

[27] R Core Team. R: A language and environment for statistical computing. R Foundation for Statistical Computing, Vienna, Austria, 2018. https://www.R-project.org/.

[28] Alvergne A. EHM_Alvergnetal_Q1_aggregate_data. figshare. Dataset. doi:10.6084/m9.figshare.6820796.v1.

[29] Tomiyama AJ, Hunger JM, Nguyen-Cuu J et al Misclassification of cardiometabolic health when using body mass index categories in NHANES 2005–2012. Int J Obes 2016; 40:883–6. 10.1038/ijo.2016.17 26841729

[30] Pinheiro J, Bates D, DebRoy S, Sarkar DR Core Team (2018) _nlme: Linear and Nonlinear Mixed Effects Models_. R package version 3.1–137, https://CRAN.R-project.org/package=nlme.

[31] Gelman A, Hill J. Data Analysis Using Regression and Multilevel/Hierarchical Models. New York: Cambridge University Press, 2007.

[32] Leifeld P texreg: Conversion of Statistical Model Output in R to LaTeX and HTML Tables. Journal of Statistical Software 2013; 55:1–24.

[33] Reed BG, Carr BR et al The normal menstrual cycle and the control of ovulation In: De Groot LJ, Chrousos G, Dungan K, *et al.* (eds.). Endotext [Internet]. South Dartmouth (MA): MDText.com Inc, 2000.

[34] Doyle C, Ewald HA, Ewald PW. Premenstrual syndrome: an evolutionary perspective on its causes and treatment. Perspect Biol Med 2007; 50:181–202. 17468538 10.1353/pbm.2007.0015

[35] Lesnoff M, Lancelot R. *Aod: Analysis of Overdispersed Data*, 2012.

[36] Jasienska G, The Fragile Wisdom. Harvard University Press, 2013.

[37] King AE, Critchley HOD. Oestrogen and progesterone regulation of inflammatory processes in the human endometrium. J Steroid Biochem Mol Biol 2010; 120:116–26. 20067835 10.1016/j.jsbmb.2010.01.003

[38] Centers for disease control and prevention. Human Papillomavirus (HPV): Genital HPV Infection – Fact Sheet. https://www.cdc.gov/std/hpv/stdfact-hpv.htm (17 July 2018, date last accessed).

[39] Canli T. Reconceptualizing major depressive disorder as an infectious disease. Biol Mood Anxiety Disord 2014; 4:10. 25364500 10.1186/2045-5380-4-10 PMC4215336

[40] Maier SF. Bi-directional immune-brain communication: implications for understanding stress, pain, and cognition. Brain Behav Immun 2003; 17:69–85. 12676570 10.1016/s0889-1591(03)00032-1

[41] Aiyar A, Quayle AJ, Buckner LR et al Influence of the tryptophan-indole-IFNγ axis on human genital Chlamydia trachomatis infection: role of vaginal co-infections. Front Cell Infect Microbiol 2014; 4:72. 24918090 10.3389/fcimb.2014.00072 PMC4042155

[42] Michel KG, Huijbregts RPH, Gleason JL et al Effect of hormonal contraception on the function of plasmacytoid dendritic cells and distribution of immune cell populations in the female reproductive tract. J Acquir Immune Defic Syndr 2015; 68:511–8. 25763784 10.1097/QAI.0000000000000531 PMC4874780

[43] Bancroft J, Rennie D. The impact of oral contraceptives on the experience of perimenstrual mood, clumsiness, food craving and other symptoms. J Psychosom Res 1993; 37:195–202. 8463994 10.1016/0022-3999(93)90086-u

[44] Graham CA, Sherwin BB. A prospective treatment study of premenstrual symptoms using a triphasic oral contraceptive. J Psychosom Res 1992; 36:257–66. 1564678 10.1016/0022-3999(92)90090-o

[45] Roger S L, David MG, Gretchen ML et al Comprehensive Gynecology. Philadelphia: Elsevier, 2016.

[46] Ussher JM, Perz J, May E. Pathology or source of power? The construction and experience of premenstrual syndrome within two contrasting cases. Fem Psychol 2014; 24:332–51.

[47] Campagne DM, Campagne G. The premenstrual syndrome revisited. Eur J Obstet Gynecol Reprod Biol 2007; 130:4–17 16916572 10.1016/j.ejogrb.2006.06.020

[48] Henrich J, Heine SJ, Norenzayan A. Most people are not WEIRD. Nature 2010; 466:29–29. 20595995 10.1038/466029a

[49] Jasienska G, Jasienski M. Interpopulation, interindividual, intercycle, and intracycle natural variation in progesterone levels: A quantitative assessment and implications for population studies. Am J Hum Biol 2008; 20:35–42 17963226 10.1002/ajhb.20686

