# Supplementary material for: Do sexually transmitted infections exacerbate negative premenstrual symptoms? Insights from digital health
Source: Evol Med Public Health. 2018 Jul 3;2018(1):138–50. doi: 10.1093/emph/eoy018 (PMC6070031; doi:10.1093/emph/eoy018)
Supplement: Supplementary Data [file eoy018_sifigure.docx]

Do sexually transmitted infections exacerbate negative premenstrual symptoms? Insights from digital health

Alexandra Alvergne^1^, Marija Vlajic Wheeler^2^, Vedrana Högqvist Tabor^3,4^

**Supplementary Information**

**Figure SI1**


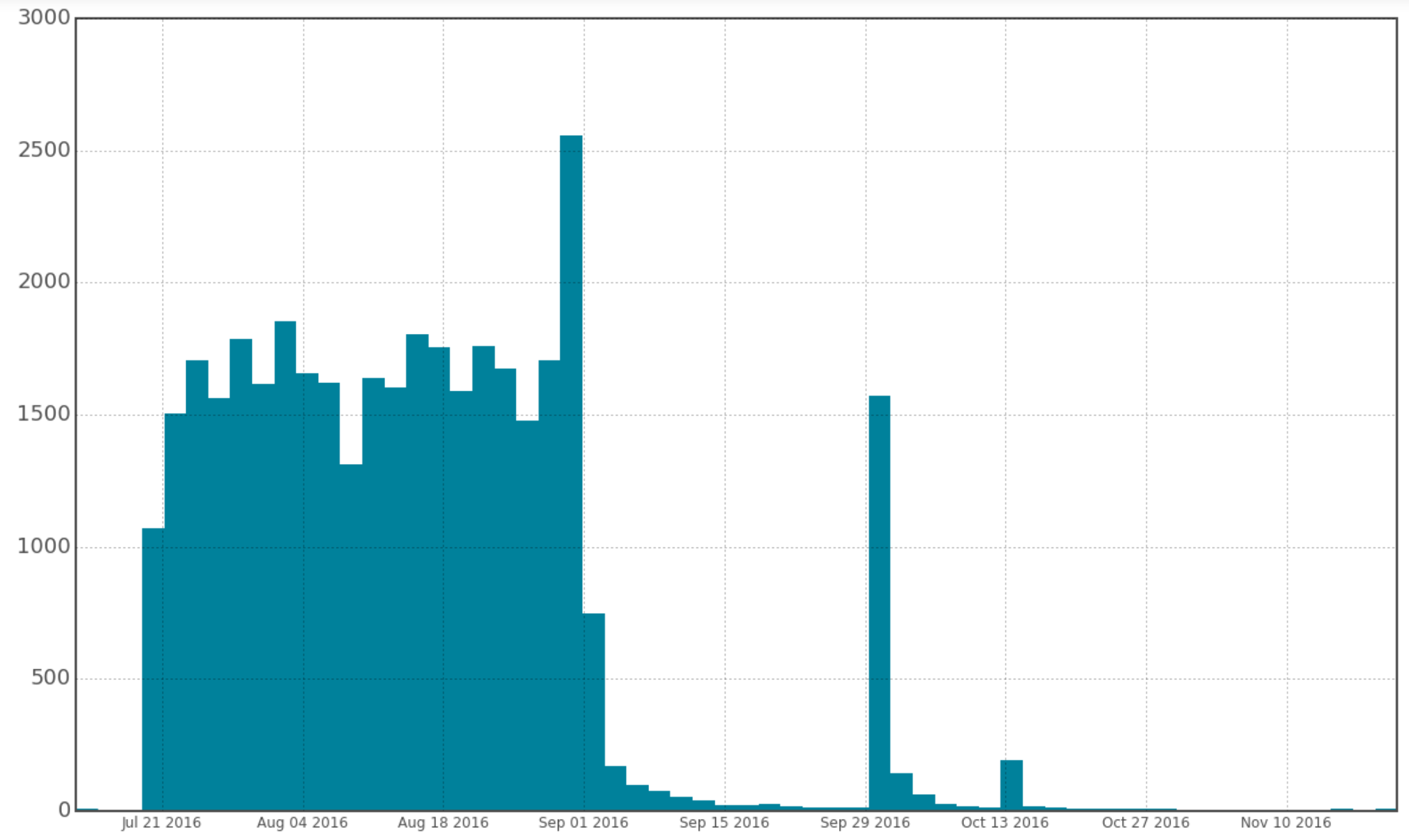
**Figure SI1: Distribution of responses over time.** The first responses came in on the 19/07/16 and the survey was stopped exactly 3 months later on the 19/10/16. The peak in September corresponds to a “push” of the survey at a *Clue* ambassador event.
